# Barcoded Orthotopic Patient‐Derived Head & Neck Squamous Cell Carcinoma Model Demonstrating Clonal Stability and Maintenance of Cancer Driver Mutational Landscape

**DOI:** 10.1002/cam4.71137

**Published:** 2025-08-12

**Authors:** Peiran Zhou, Claire B. Mills, Zhao Ming Dong, Brittany R. Barber, Slobodan Beronja

**Affiliations:** ^1^ Human Biology Division Fred Hutchinson Cancer Center Seattle Washington USA; ^2^ Department of Otolaryngology—Head and Neck Surgery University of Washington Seattle Washington USA; ^3^ US Department of Veterans Affairs, VA Puget Sound Health Care System Seattle Washington USA

**Keywords:** clonal stability, genetic landscapes, high engraftment rate, HNSCC, orthotopic PDX

## Abstract

**Objective:**

To illustrate a new barcoded orthotopic patient‐derived xenograft (PDX) mouse model where one can investigate phenotypic effects of single‐cell level gene manipulation in a pooled format. To address some concerns of current PDX mouse models of head and neck squamous cell carcinoma (HNSCC): (1) genomic evolution with passage by generating high‐purity cancer cells, which can also be utilized for other downstream applications, including cell culture‐based studies, and (2) cost‐effectiveness of current PDX models.

**Methods:**

Two‐millimeter tumor cubes from nine patients were implanted into immunodeficient mouse flanks subcutaneously. Purified tumor cells were obtained from subcutaneous xenografts. Various numbers of purified tumor cells were then injected into the lingual tissue of immunodeficient mice, and the lowest amount of cells needed to achieve a 100% orthotopic engraftment rate were identified. Clonal stability was tested using a lentiviral barcoding system. The orthotopic PDXs' genetic landscapes were characterized using whole exome sequencing.

**Results:**

This approach yielded an overall engraftment rate of 88.9%. The purification process increased cancer cell purity from 34% to 92%. Lingual injection of 100,000 purified tumor cells achieved a 100% orthotopic engraftment rate from purified subcutaneous PDX tumor cells while maintaining clonal and genetic stability.

**Conclusion:**

Our study presents a barcoded orthotopic patient‐derived xenograft model for head and neck squamous cell carcinoma with clonal stability. This model provides a way to study phenotypic effects of single cell level gene manipulation in a pooled format. The method can be adapted for in vitro work as well.

## Graphical abstract

Barcoded orthotopic HNSCC PDX with high engraftment rate, clonal stability, and its genetic landscapes as an alternative method to obtain high quantities of purified tumor cells for downstream work.

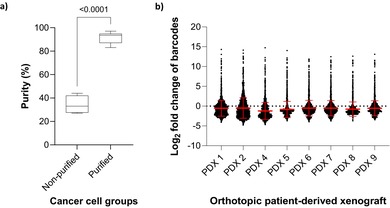

## 1 Introduction

Insights into the biological basis and therefore development of better approaches to head and neck squamous cell carcinoma (HNSCC) therapy have been facilitated via judicious use of cell and tissue models that reproduce key features of human tumors [1]. Isolation of the first HNSCC cell lines can be traced back to the 1950s [2]. Since then, more HNSCC cell lines have been established [1, 3, 4, 5, 6]. In order to model the complex interactions between the cancer and its host, cell line‐derived xenografts in mice have been developed [7]. Since then, various in vitro and in vivo preclinical models have been introduced: from immortalized cell lines to 3D organoids, from patient‐derived xenografts (PDX) to carcinogenic‐induced or transgenic animal models. In vitro models in general are cost‐effective, easy to maintain, and grow rapidly. However, they also carry a set of disadvantages, such as misidentification and cross‐contamination [6]. The immortal HNSCC cell lines often carry little resemblance to the HNSCC tumors, and vice versa [7]. Three‐dimensional spheroids/organoids are limited by the lack of extracellular matrix and atypical physiology [8]. In addition, cell culture can induce significant clonal selection and genomic instability [9]. On the other hand, in vivo models have the advantages of better resembling the tumor microenvironment and human disease [1, 9], including its histopathologic features, genetics, epigenetics, and response to treatments [10]. However, there is concern about subclonal expansion and genomic evolution with each passage of current PDX models. Therefore, later generations of the PDX may not accurately reflect the original patient tumor [9, 11, 12]. In addition, the PDX model is also limited by its relatively higher cost [13]. Thus, identifying optimized conditions to generate orthotopic PDX models to better reflect the mutational and clonal complexity of HNSCC tumors and improve current PDX models to allow investigation of phenotypic effects in a pooled format within one PDX mouse remains a critical task.

This study presents an alternative method to obtain highly purified HNSCC cells using a PDX model without flow cytometry, cell sorting, or multiple cell passages [14, 15]. These purified cells can be used in a variety of downstream applications, including studying mechanisms of cancer biology, therapeutic testing, and developing in vitro models, in which contamination with cancer‐associated fibroblasts presents a challenge. Using these purified cancer cells, we demonstrated the optimal number of cancer cells for orthotopic PDX establishment, with a high engraftment rate while maintaining the stability of each cancer cell population. We further demonstrated a novel single cell lentiviral barcoding system to track the behavior of transplanted HNSCC PDXs over time and demonstrate minimal clonal drift. We characterized the genetic mutational landscape of our PDXs compared to that of HNSCC tumor samples to demonstrate the maintenance of core driver lesions across our model. Finally, we correlated the PDX tumor heterogeneity and its genetic landscape stability. We expect our method to be adopted as a key step in future studies of HNSCC, including in small‐ and large‐scale functional assays that may introduce additional genetic modifications and monitor downstream effects in one setting, in both culture‐based and animal‐based models.

## 2 Methods

This study was approved by the Fred Hutchinson Cancer Center Institutional Review Board (IRB 10571). Prospective tumors were screened prior to resection. New and recurrent head and neck mucosal SCC patients who underwent surgical resection were included. HPV+ patients (per P16+ staining) were excluded. Written informed consents were obtained from all participants. To ensure unaffected clinical care of all participants, excess, non‐diagnostic fresh tumor tissue samples and blood samples were obtained on the day of surgical resection via University of Washington Northwest BioTrust. Patients were enrolled from September 2021 to June 2022.

Immunocompromised NOD/LtSz‐*Prkdc* ^*scid*^ *Il2rg* ^*tm1Wjl*^/J (NSG) mice used in the study were housed and cared for in an Association for Assessment and Accreditation of Laboratory Animal Care (AAALAC)—accredited facility at the Fred Hutchinson Cancer Center. All animal experiments were conducted in accordance with the Fred Hutchinson Cancer Center ethical regulations and Institutional Animal Care & Use Committee (IACUC)—approved protocols (project license number 50814).

Hematoxylin and eosin (H&E) slides were prepared for both patient tumor specimens and corresponding PDX tissues. Formalin‐fixed, paraffin‐embedded (FFPE) tissue blocks were sectioned at 5 μm thickness using a microtome and mounted onto positively charged glass slides. Sections were deparaffinized in xylene, rehydrated through graded ethanol, and stained with hematoxylin for nuclear visualization followed by eosin for cytoplasmic contrast. Stained slides were dehydrated, cleared, and cover slipped using a standard mounting medium. The slides were reviewed by board‐certified pathologists to confirm histologic diagnosis and compare histologic grade between patient and PDX samples.

Freshly resected patient tumors were preserved in Dulbecco's Modified Eagle Medium (DMEM; Thermo Fisher Scientific, Waltham, MA) supplemented with 100 U/mL penicillin and 100 μg/mL streptomycin (Gibco Penicillin‐Streptomycin; Thermo Fisher Scientific). These samples were transported to the laboratory on ice within 45 min of resection. Five NSG mice per patient sample were grafted with tumor tissue subcutaneously on their flank, with each mouse receiving a ~2 mm × 2 mm tissue block. The time from tissue arrival in the laboratory to the completion of subcutaneous transplantation was also documented. The subcutaneously engrafted tumors were harvested when they reached a size of approximately 1.5 cm in diameter. Following harvest, single cancer cell solutions were prepared through scalpel mincing, followed by enzymatic digestion and mechanical dissociation using the gentleMACS Octo Dissociator (Miltenyi Biotec, Bergisch Gladbach, Germany) and the Tumor Dissociation Kit, Human (Miltenyi Biotec). The purification process was carried out using the Mouse Cell Depletion Kit (Miltenyi Biotec). Various numbers of purified cancer cells in a 20 μL volume were injected into the lingual tissues of NSG mice, and the orthotopic engraftment rates were documented.

A lentiviral barcode system modified from an existing in utero lentiviral injection method [16, 17], based on a thoroughly validated principle [18, 19, 20], was utilized to assess clonal stability and demonstrate the traceability of cancer cells within the PDX models (detailed lentiviral barcode generation and methods are in Data S1 under “Lentiviral barcode system”). In brief, a pool of 2000 genetic barcodes, each composed of 10 nucleotides along with genetic code for red fluorescence protein (RFP) marker, was transduced into cancer cells via lentiviral infection. The infection rate was intentionally set at approximately 15% to ensure a multiplicity of infection (MOI) ≤ 1, meaning that each cancer cell would receive a maximum of one barcode. The infection rate and cell viability were measured with flow cytometry. The transduction was carried out at a cancer cell concentration of 500,000 cells per mL and a lentivirus concentration calibrated to achieve the target infection rate within 4 h, thereby ensuring optimal cancer cell viability (see Data S1). This process was conducted in cell culture media, comprising DMEM (Thermo Fisher Scientific) supplemented with 10% Fetal Bovine Serum (FBS, Thermo Fisher Scientific), 100 U/mL penicillin, and 100 μg/mL streptomycin (Gibco Penicillin‐Streptomycin; Thermo Fisher Scientific), and it took place in Corning Costar Ultra‐Low Attachment Multiple Well Plates (Corning, Corning, NY) at 37°C with 5% CO_2_ with gentle pipette mixing every 30 min. A fraction of the freshly transduced cancer cells (~1,500,000) was set aside to quantify individual barcode abundance at *t* = 0, while the remainder was injected into the tongues of NSG mice, with each mouse receiving 100,000 cancer cells in a 20 μL volume of PBS. Upon reaching a diameter of 6 mm, the lingual tumors were resected, and genomic DNA was isolated using DNeasy Blood & Tissue Kit (Qiagen, Hilden, Germany). Subsequently, the barcode region was amplified through PCR, and the amplicon was isolated following agarose gel electrophoresis. Barcode abundance at *t* = 0 and in tumor samples was quantified using NGS and DESeq2 analysis methods [21], a widely recognized and robust statistical approach for assessing differential gene expression, as previously described [18], on Galaxy platform [22].

To characterize the genetic mutation landscape of our PDX models, the mutational profiles of orthotopic PDX cancer cells were compared to those of patient tumor tissue using whole exome sequencing (WES), with normalization to peripheral blood samples to account for germline mutations. Genomic DNA was extracted using the DNeasy Blood & Tissue Kit (Qiagen). Next generation sequencing (NGS) library preparation was carried out using the Twist 96‐Plex Library Prep Kit (Twist Bioscience, South San Francisco, CA). Library quality control and quantification were performed using TapeStation 4200 (Agilent Technologies Santa Clara, CA) and Qubit 4 Fluorometer (Invitrogen, Waltham, MA). Libraries were sequenced utilizing the NovaSeq 6000 SP (Illumina, San Diego, CA).

For data analysis, NGS adapters were trimmed from paired‐end reads using cutadapt [23] and trimmed reads were mapped to reference genome hg38 using BWA 0.7.17 to obtain whole exome sequencing (WES) data [24]. Somatic mutations were called using MuTect2. VCF files were annotated using VEP. Only MuTect2 calls marked as “PASS” were selected. Mutations with a low variant allele frequency (< 0.02) were removed. Intronic mutations, mutations in the UTR or UTR‐flanking regions, and silent mutations were also removed. Both full mutation profiles as well as driver mutations were compared between primary tumor and PDX. The list of 133 HNSCC driver mutations was curated from different sources including TCGA and IntOGen [25, 26, 27, 28, 29, 30]. Fraction and Allele‐Specific Copy Number Estimates from Tumor Sequencing (FACETS) [31] was utilized to calculate the sample purity.

## 3 Results

### 3.1 Patient Characteristics

**FIGURE 1:**
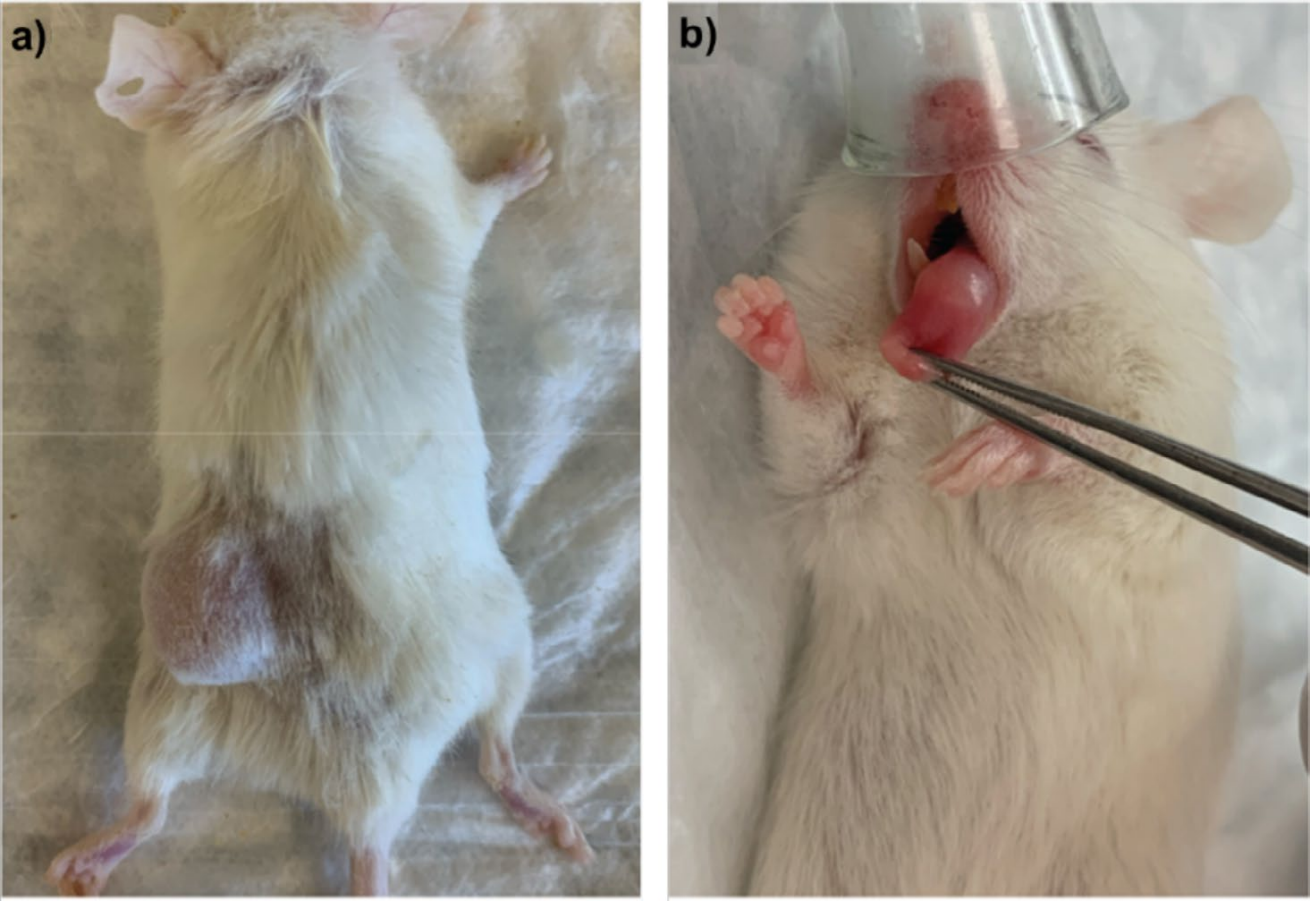
(a) head and neck squamous cell carcinoma (HNSCC) patient‐derived xenograft tumor with subcutaneous tissue implant. (b) HNSCC patient‐derived xenograft tumor with lingual injection.

A comprehensive dataset of patient characteristics and PDXs generation status is presented in Table 1. The table provides information regarding patient age, sex, tobacco use history, and alcohol use history. Additionally, tumor staging according to the 8th edition AJCC staging system, histological grade, along with the specific cancer sites, is included. The average age ± standard deviation is 62.7 ± 16.1, with 55.6% being male. TNM staging ranged from II to IVb. Exposure history to tobacco and alcohol ranged from non‐tobacco/alcohol user to heavy tobacco/alcohol user. A representative subcutaneous and lingual PDX tumor is shown in Figure 1.

**TABLE 1:** Patient characteristics and patient‐derived xenograft (PDX) engraftment status.

Patient id	Age	Sex	Site	TMN stage	Histological grade	Tobacco exposure	Alcohol exposure	PDX Engrafted/Id
Pt1	31	M	Tongue	pT4aN3bM0	G2	Nonsmoker	Socially	Yes/PDX1
Pt2	60	F	BOT	pT3N3bM0	G2	50 pack year	1 drink/day	Yes/PDX2
Pt3^a^	66	M	Larynx	pT3N0M0	G2	Nonsmoker	1 drink/week	No/NA
Pt4	81	F	Tongue	pT4aN2aM0	G2	10 pack year	Socially	Yes/PDX4
Pt5	66	M	Larynx	pT4aN2aM0	G3	10 pack year	Heavily	Yes/PDX5
Pt6	57	F	Tongue	pT2N0M0	G2	Nonsmoker	Socially	Yes/PDX6
Pt7^b^	71	M	Gingiva	pT4aN0M0	G1	10 pack year	Quit, heavily	Yes/PDX7
Pt8	87	F	Tongue	pT1N2aM0	G2	Nonsmoker	None	Yes/PDX8
Pt9^c^	46	M	Tongue	pT4aN0M0	G1	1 pack year	Socially	Yes/PDX9

^a^ History of oropharyngeal SCC underwent chemoradiation.

^b^ History of tongue SCC underwent resection.

^c^ History of tongue SCC underwent resection.

### 3.2 PDX Engraftment Rate and Processing Time

We employed an initial subcutaneous engraftment strategy prior to orthotopic implantation to maximize the utility from limited and valuable patient tumor specimens. Subcutaneous implantation requires minimal tissue processing, thereby reducing cell loss during handling. The procedure for subcutaneous engraftment is significantly shorter in duration (approximately 40 min vs. 200 min for lingual orthotopic engraftment), minimizing time ex vivo. Additionally, subcutaneous tumors can grow to larger sizes without necessitating animal sacrifice, enabling the expansion of tumor cells from first‐generation PDXs for downstream applications, including orthotopic modeling.

Subcutaneous and subsequent orthotopic engraftments were seen in eight out of nine patients (88.89%). Patient tumor samples were first implanted into the flanks of NSG mice. Once those reached 1.5 cm in diameter size, they were then dissociated, purified, and implanted into tongues to better recapitulate the cells' native environment. One patient (11.11%) with stage III laryngeal SCC did not generate any xenograft 6 months after implantation. Through serial dilutions and injections, we determined that 100,000 purified tumor cells injected into the lingual tissue of NSG mice was the minimum number of tumor cells needed to achieve 100% subsequent orthotopic engraftments from subcutaneous xenografts (The cell number injected and corresponding orthotopic engraftment rates are shown in Table S1).

The patient tumor handling time, from the arrival of cancer tissue in the laboratory to completion of the subcutaneous flank engraftment procedure into mice was 40 ± 4 min. Latency periods were also recorded for orthotopic PDX models, and all PDXs developed within 7 weeks; mice receiving tumor tissue from the same patient developed engraftment within 2 weeks of each other.

### 3.3 H&E Slides of Original Tumor and Orthotopic PDX Tumor

**FIGURE 2:**
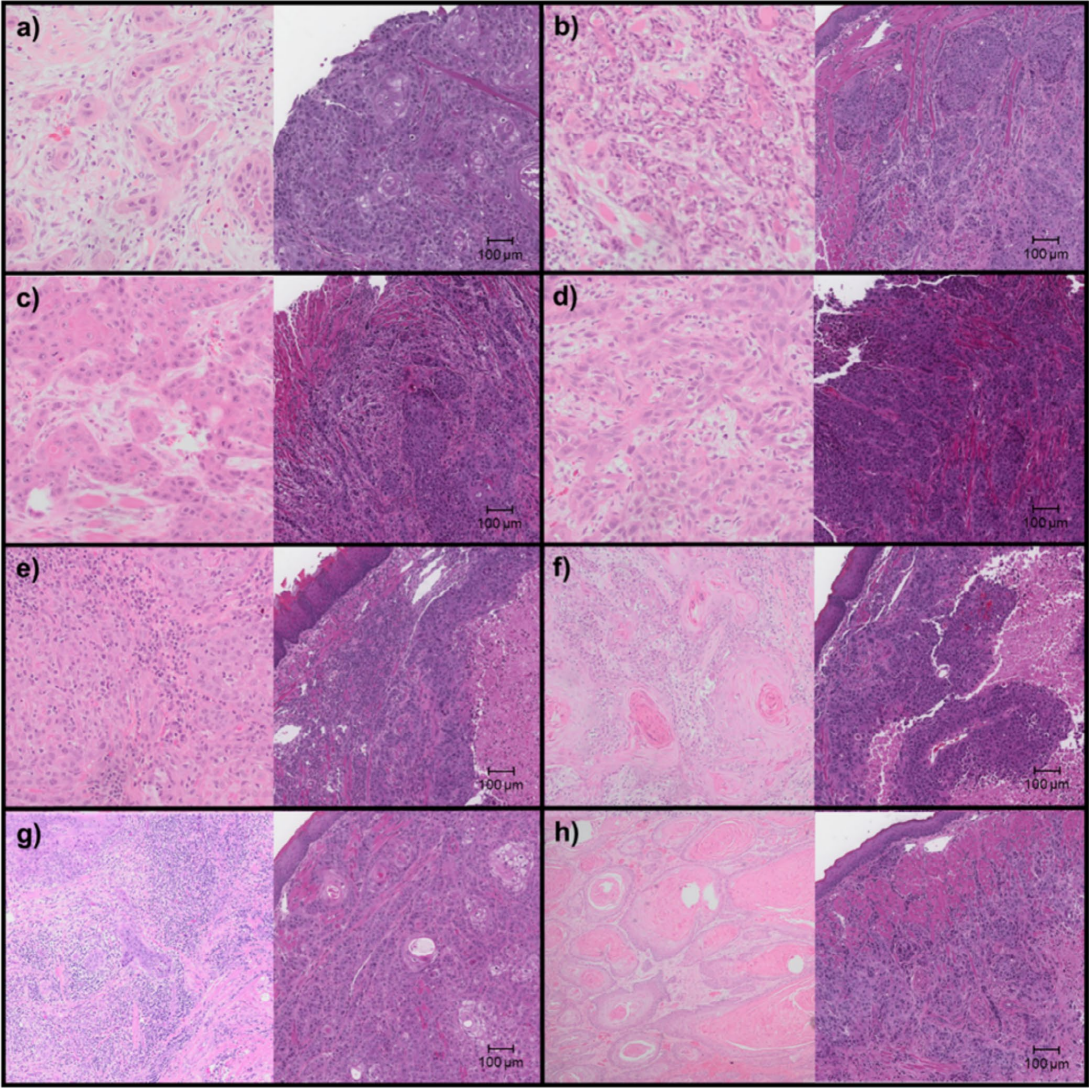
H&E slides showing head and neck squamous cell carcinoma (HNSCC) of patient tumor (left) and corresponding orthotopic patient‐derived xenograft (PDX) (right) of (a) Pt1 & PDX1, (b) Pt2 & PDX2, (c) Pt4 & PDX4, (d) Pt5 & PDX5, (e) Pt6 & PDX6, (f) Pt7 & PDX7, (g) Pt8 & PDX8, and (h) Pt9 & PDX9.

The Representative H&E slides of patient tumor and corresponding PDX tumor are shown in Figure 2. The histological grades for patient tumors are detailed in Table 1. The corresponding PDX H&E slides were reviewed and graded as G3 for PDX5 and G2/G3 for the other 7 PDX tumors. This is consistent with prior observation that a higher histopathological grade was strongly associated with passage, while maintaining genetic stability [32].

### 3.4 PDX Tumor Cell Purification

**FIGURE 3:**
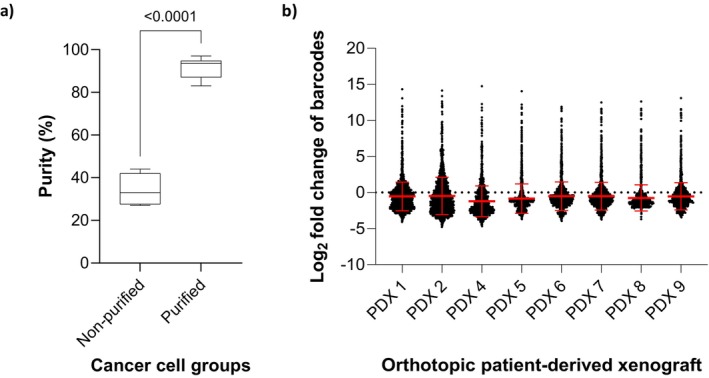
(a) Mean and standard deviation of cancer cell purity in single cell suspension without and with purification. (b) Log2 fold changes in barcode abundance of orthotopic patient‐derived xenograft (PDXs), tumor harvest when they reached 6 mm in diameter.

Host stromal cell contamination in PDX tumors can compromise the depth of downstream genomic sequencing and hinder downstream in vitro models and studies. Fluorescence‐activated cell sorting (FACS) is commonly used to isolate the target cell population. However, this technique is expensive, time consuming, and can negatively affect cell viability [33]. To streamline the process to eliminate murine cell contamination and isolate purified human tumor cells from initial subcutaneous engraftments, we employed the Miltenyi Mouse Cell Depletion Kit. In brief, once the subcutaneous PDX tumors reached 1.5 cm, the tumors were resected, and single cell suspensions were generated using the Miltenyi gentleMACS Octo Dissociator and the Tumor Dissociation Kit. A portion of the pre‐purified cells was saved for comparison. The remaining single cell suspensions were purified using the Mouse Cell Depletion Kit. All kits were used according to the manufacturer's protocol. Genomic DNA was extracted from pre‐ and post‐purified samples, and WES was performed as described above. FACETS [31] was utilized to calculate tumor purity (an example of FACETS output was shown in Figure S2 and purity summarized in Table S2). As illustrated in Figure 3a, the utilization of the mouse depletion kit led to a significant enhancement in the purity of cancer cells within the single cell suspension from the subcutaneous xenografts. Prior to purification, the cancer cell purity stood at 34% (95% CI: 28%–41%). However, following the application of the kit, the purity remarkably increased to 92% (95% CI: 88%–95%), demonstrating a substantial improvement with a highly significant *p*‐value < 0.0001. In addition to generating orthotopic PDXs described above, the purified tumor cells were successfully cultured up to 5 days after lentiviral infection to allow expression of RFP to determine the infection rate without fibroblast contamination. We did not pursue further cell passage to develop cell lines as this was not the focus of the current work.

### 3.5 Cancer Cell Clonal Stability and Diversity in PDX

We identified that lingual injections of 100,000 purified tumor cells from subcutaneous PDX tumors into immunodeficient mouse tongues yielded a 100% orthotopic engraftment rate in this study (see Data S1 “Cell number and engraftment rate”). Subclonal expansion in cancer models has been suggested and attributed as reason for genetic drift [11]. A lentiviral barcode system was utilized to track the clonal changes and assess the clonal stability in orthotopic PDX model. In brief, purified tumor cells were transduced with lentivirus as described in the method section. Each lentivirus contains one unique 10‐basepair barcode, and a pool of 2000 distinct barcodes were used to adequately assess clonal stability over time. The infection rate was set at approximately 15% to ensure a MOI ≤ 1, meaning that each cancer cell would receive a maximum of one barcode. The infection rate was measured with flow cytometry using RFP as a marker. To ensure > 100× coverage of the barcodes at 15% infection rate, 1,500,000 infected cells were saved to measure the barcodes distribution at *T* = 0 and 15 orthotopic PDXs (100,000 cells per PDX at total of 1,500,000 cells) were generated to measure the barcodes distribution when tongue tumors reached 6 mm. Genomic DNA was extracted from the samples and barcode specific regions were amplified using PCR. The amplicons were separated using gel electrophoresis and corresponding bands were isolated and sequenced. Barcode abundance at *t* = 0 and in tumor samples was quantified using NGS and DESeq2 analysis methods [21]. The change of lentiviral barcode distribution in orthotopic PDX tumors compared to the distribution measured at *T* = 0, calculated as log_2_ change is shown in Figure 3b in the format of a violin plot: cluster of log_2_ change for each barcode. Notably, all barcodes maintained within the PDXs at 100× barcode coverage (15 PDX pooled together), without significant abundance changes. This suggests consistent clonal stability in the PDX models, demonstrating the injection of 100,000 purified tumor cells as the optimal condition for ensuring a high engraftment rate while preserving clonal stability. However, this does not necessarily mean that all 100,000 tumor cells injected into one mouse will develop into PDX tumor.

To assess clonal diversity within samples, we calculated both the Shannon and Simpson diversity indices, which are commonly used ecological diversity measures adapted for barcode diversity analysis. The Shannon diversity index accounts for both richness (number of unique barcodes) and evenness (relative abundance). Higher values indicate greater diversity, with typical values ranging from 0 (no diversity) to 4 for highly diverse populations [34]. The Simpson diversity index emphasizes dominance and the probability that two randomly selected barcodes belong to different clones. This index ranges from 0 to 1, with values closer to 1 indicating greater diversity [35]. Compared to the Shannon index, Simpson's index is more sensitive to changes in the most abundant clones. These diversity indices of PDX models are shown in Table 2.

**TABLE 2:** Diversity indices of barcode in patient‐derived xenograft (PDX) tumors.

Sample id	Shannon index	Simpson's index
PDX1	3.7077	0.9433
PDX2	3.4425	0.9250
PDX4	3.4158	0.9308
PDX5	3.5865	0.9457
PDX6	3.2006	0.9115
PDX7	3.4417	0.9314
PDX8	3.0455	0.9001
PDX9	2.6385	0.8038

### 3.6 Genomic Mutation Landscape of PDX

**FIGURE 4:**
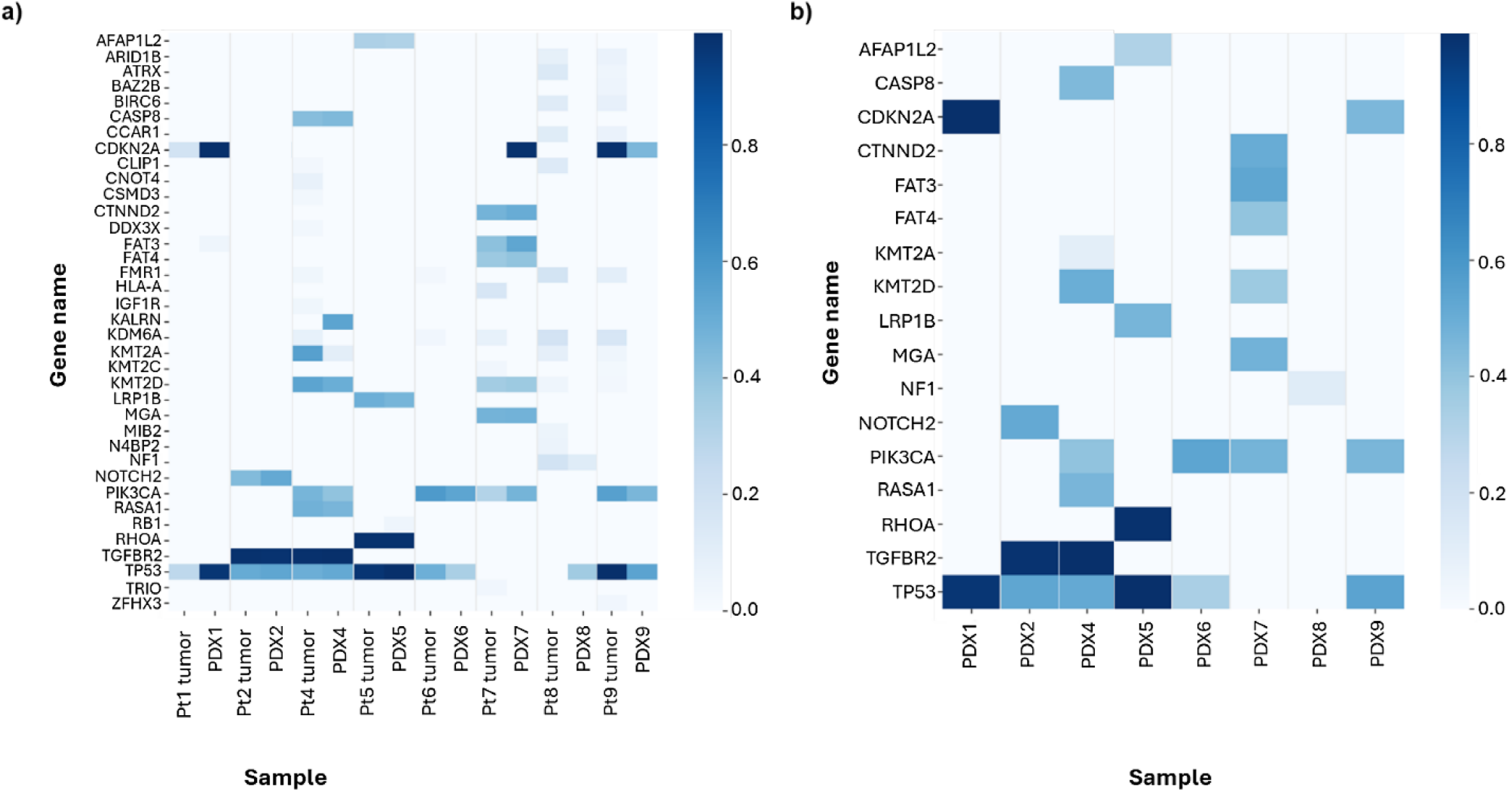
(a) Heatmap of driver gene allele frequencies in patient tumors and corresponding patient‐derived xenograft (PDX) tumors. (b) Heatmap of driver gene allele frequencies retained in PDX tumors.

The driver gene allele frequencies in patient tumors and corresponding PDX tumors and the driver genes retained in PDX tumors are shown in Figure 4a,b, respectively. The genetic mutational landscape of patients' primary tumors and corresponding PDX tumors is summarized in Table 3. The tumors used in our study showed common drivers in HNSCC [25, 26, 27, 28, 29, 30]. The PDX tumors retained the majority of the driver mutations in primary patient tumors and showed a similar driver mutation landscape. Three PDXs (PDX2, PDX6, and PDX9) maintained all original drivers, and five PDXs lost one original driver. PDX4, PDX6, PDX7, PDX8, and PDX9 gained additional driver mutations, while PDX1, PDX2, and PDX5 did not. Five out of eight PDXs gained a significant amount of passenger mutations when all exonic mutations were compared. The percentage of all mutations maintained in PDX tumors ranges from 30% to 89%.

**TABLE 3:** Genetic mutational landscape of patients' tumor and corresponding patient‐derived xenograft (PDX).

Patient id	# of mutation	PDX id	# of mutation	Overlapping mutation	% mutation maintained
**All exonic mutations**					
Pt1	56	PDX1	28	23	41
Pt2	87	PDX2	74	61	70
Pt4	106	PDX4	399	89	84
Pt5	199	PDX5	180	167	84
Pt6	67	PDX6	191	47	70
Pt7	140	PDX7	299	124	89
Pt8	61	PDX8	706	18	30
Pt9	53	PDX9	655	42	79
**Driver Mutations**					
Pt1	3	PDX1	2	2	67
Pt2	4	PDX2	4	4	100
Pt4	9	PDX4	18	8	89
Pt5	5	PDX5	4	4	80
Pt6	2	PDX6	6	2	100
Pt7	8	PDX7	13	7	88
Pt8	2	PDX8	18	1	50
Pt9	3	PDX9	21	3	100

### 3.7 Effect of Genomic Landscape Change on Clonal Diversity

**FIGURE 5:**
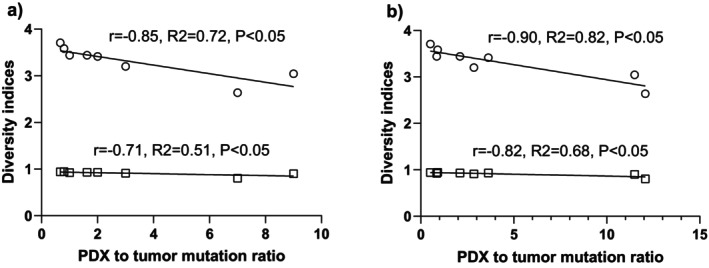
The patient‐derived xenograft (PDX) barcode diversity indices (Shannon index in open circle and Simpson's index in open square) versus the ratio of PDX mutation to tumor mutation of (a) driver mutation and (b) total mutation.

To investigate the relationship between clonal diversity and mutational divergence in PDXs, we compared barcode diversity indices with the ratio of PDX to tumor mutations. As shown in Figure 5, both Shannon and Simpson diversity indices negatively correlated with the mutation ratio, suggesting that the expansion of highly fit subclones with new mutations reduced clonal diversity. Specifically, in Figure 5a, which examines driver mutations, the Shannon index showed a strong negative correlation (*r* = −0.85, *R* ^2^ = 0.72), while the Simpson index also demonstrated a significant inverse relationship (*r* = −0.71, *R* ^2^ = 0.51). A similar trend was observed when analyzing total mutations (Figure 5b), with even stronger correlations: Shannon index *r* = −0.90 (*R* ^2^ = 0.82) and Simpson index *r* = −0.82 (*R* ^2^ = 0.68). These data indicate that clonal dominance in PDX models is associated with the acquisition of new mutations, supporting the hypothesis that such mutations confer a selective advantage and lead to reduced clonal diversity.

## 4 Discussion

There are several commonly used patient‐derived preclinical models, including immortalized cell lines, organoids, and xenografts. Once generated, the immortal cell lines are cost‐effective, easy to maintain, and quick to establish. However, culture‐based models can induce significant clonal selection and genomic instability [9], often carrying little resemblance to the HNSCC tumors [7]. Patient‐derived organoids share the advantages of other in vitro models. They have gained traction in recent years due to their ability to allow for high‐throughput drug testing while recapitulating key aspects of tumor architecture and genetics [36, 37]. However, they are limited by the lack of extracellular matrix and atypical physiology [8]. In addition, the organoid establishment rates for head and neck cancers are low, with one large‐scale study reporting a success rate of approximately 30% [37].

Since the conception of PDX over 70 years ago [38], it has become an important preclinical model to study cancer biology and anti‐cancer therapies [10]. PDX models have the advantages of better resembling the tumor microenvironment and human disease [1, 9], including its histopathologic features, genetics, epigenetics, and response to treatments [10]. The PDX models can be subdivided into heterotopic (flank) models and orthotopic (buccal mucosa or lingual) models. However, there is concern of subclonal expansion and genomic evolution with each passage of current PDX models; therefore, later generations of PDX may not accurately reflect the original patient tumor [9, 11, 12]. In addition, PDX generation is more expensive to develop and maintain, and orthotopic HNSCC is considered technically challenging [39]. It is currently unknown whether orthotopic PDX significantly differs from heterotopic PDX in terms of growth rate and treatment response [40]. However, the orthotopic PDX provides a means to study lymph node metastasis [40, 41].

This study presents a barcoded orthotopic PDX model for HNSCC that allows the investigation of phenotypic effects of single cell level gene manipulation in a pooled format, such as identifying critical genetic events driving the transition from dysplasia to carcinoma and evaluating targeted therapies. This can significantly reduce the cost of utilizing PDX models. We also presented solutions to address some concerns of current PDX mouse models of HNSCC: (1) concerns of genomic evolution with each passage by generating high purity cancer cells from the initial PDX tumor and (2) cost effectiveness of PDX by first implanting in flanks of mice, then purifying cells and implanting sublingually. This study established a pipeline for generating orthotopic HNSCC PDX models, which has the advantage of mimicking the tumor's natural environment and was previously deemed to be difficult due to procedural challenges [39]. Our approach achieved a high engraftment rate of 88.89%, which is at the upper end of the reported HNSCC PDX engraftment rates [42, 43, 44, 45]. The processing time was 40 ± 4 min, which is at the lower end of reported sample processing time, ranged from immediately following resection to 24 h [46, 47, 48, 49, 50, 51]. The short processing time and minimal handling of the tumor sample are likely contributors to the high engraftment rate [45]. In addition, NSG mice, which have a full null mutation in the interleukin‐2 gene resulting in innate immunity deficiency [52], are the most efficient mouse strain for engraftment of the primary human tumor [53, 54]. The latency time of 49 days was also in the lower range of existing PDX models (30–401 days) [45].

Subclonal expansion and genetic evolution are another concern with prior PDX models. In this study, we demonstrated an efficient method to obtain purified tumor cells and identified the minimal number of tumor cells required to achieve a high engraftment rate while maintaining clonal stability. Furthermore, we calculated the diversity indices of the PDX models and showed that they are negatively correlated with the numbers of new mutations gained in PDX tumors.

Thus far, computational purification processes have been used to analyze tumor mutational profiles due to the heterogeneous cellular populations in patient tumor samples [55, 56]. Our alternative experimental method for isolating purified cancer cells, not limited to HNSCC, offers an alternative for prognostic and treatment response predictions [55, 56, 57]. Additionally, the purified cancer cells can be utilized in various downstream processes as mentioned above. For example, the organoid generated from purified tumor cells minimized the contamination from tumor‐adjacent and non‐cancerous epithelium.

The PDX models generated in our study demonstrated their ability to faithfully represent common HNSCC driver mutations while maintaining clonal stability, consistent with prior study [58]. However, the overall mutation similarity between patient tumors and PDX tumors varied. These findings suggest the PDX models should be used with caution. Rather than assuming a PDX fully represents the original tumor, researchers should sequence its mutational profile and compare it to the original tumor, particularly in studies involving passenger mutations.

## 5 Limitations

This study is limited by small sample size, and therefore results should be interpreted with caution. The orthotopic lingual PDX model carries its intrinsic limitations such as (1) smaller size than can be achieved prior to the need to humanely euthanize the mice, (2) the technically challenging procedure for carrying out tongue injections, and (3) the ability to monitor tumor volumes accurately. In our experience, the study mice did not suffer significant weight loss until tumors reached 6 mm in diameter, and this can be easily measured under anesthesia using a periodontal probe. The lingual injection requires anesthesia as well. However, it is faster than subcutaneous implantation with less recovery time. The tumor is typically measurable when it reaches 1–1.5 mm in diameter. Intrinsic characteristics, such as cancer cell aggressiveness, are known to affect the PDX engraftment rates [45]. The participants included in this study presented with advanced disease, which may have contributed to the high engraftment rate. NSG mice are reported to be the ideal model for human tumor engraftment [53, 54]. However, the use of NSG mice limits preclinical application of PDX models as they lack representation of other components of the tumor microenvironment, including the microbiome and cancer‐immune cell interactions [59, 60]. Future investigations should prioritize the application of the established methodologies employed in this study to HNSCC PDX models in humanized mice, thereby advancing our understanding of the tumor microenvironment and providing better models for immunotherapy investigations.

## Author Contributions

**Peiran Zhou:** conceptualization (equal), data curation (equal), formal analysis (equal), funding acquisition (equal), investigation (equal), methodology (equal), writing – original draft (lead), writing – review and editing (equal). **Claire B. Mills:** data curation (supporting), formal analysis (supporting), visualization (equal), visualization (equal), writing – review and editing (equal), writing – review and editing (equal). **Zhao Ming Dong:** formal analysis (equal), visualization (equal), writing – review and editing (equal). **Brittany R. Barber:** conceptualization (equal), supervision (equal), writing – review and editing (equal). **Slobodan Beronja:** conceptualization (equal), investigation (equal), methodology (equal), supervision (equal), writing – review and editing (equal).

## Conflicts of Interest

The authors declare no conflicts of interest.

## Supporting information


**Data S1:** cam471137‐sup‐0001‐DataS1.docx.

## Data Availability

All data supporting the findings in this study are available from the corresponding authors upon request.
