# Supplementary material for: Barcoded Orthotopic Patient‐Derived Head & Neck Squamous Cell Carcinoma Model Demonstrating Clonal Stability and Maintenance of Cancer Driver Mutational Landscape
Source: Cancer Med. 2025 Aug 12;14(15):e71137. doi: 10.1002/cam4.71137 (PMC12340606; doi:10.1002/cam4.71137)
Supplement: Supplementary file 1 — Data S1: cam471137‐sup‐0001‐DataS1.docx. [file CAM4-14-e71137-s001.docx]

*Lentiviral barcode system*

HEK293T cells were used to generate barcoded lentivirus. Actively growing cells were seeded onto poly-L-lysine–coated T225 flasks at a 1:10 dilution using 40.5 mL of D10^-^ medium (DMEM supplemented with 10% FBS, 1% penicillin/streptomycin/L-glutamine, 100 mM sodium pyruvate, and 7.5% NaHCO₃). After 24 hours, a transfection mixture was freshly prepared containing 69 µg of psLenti (lentiviral vector containing RFP and barcodes), 69 µg of psPAX2 (packaging plasmid), 45 µg of pMD2.G (envelope plasmid), 570 µL of 2.5 M CaCl₂, and 2.25 mL of 2× HEPES-buffered saline (HBS), in a final volume of 4.5 mL. HBS was added last, and the solution was inverted 10 times and incubated at room temperature for 60 seconds to allow precipitate formation. The transfection mix was gently added to the culture without disturbing the cells. Granule formation was confirmed by microscopy. Twelve hours post-transfection, the medium was replaced with freshly prepared viral production medium (DMEM with 5% FBS, 1% penicillin/streptomycin/L-glutamine, 100 mM sodium pyruvate, 7.5% NaHCO₃, 0.5 M sodium butyrate, and 5 ng/mL insulin). Cells were washed twice with 2× HBS, and 45 mL of viral production medium was added. Cultures were incubated at 37°C for 24 hours. RFP expression was confirmed before harvesting. The supernatant was filtered through a 0.45 µm PES membrane (pre-moistened with DMEM), followed by filtration through a 70 kDa molecular weight cutoff (MWCO) centrifugal filter pre-washed with PBS, spun at 3,000 × g for 20 minutes. The filter was inverted to recover residual volume, then rinsed twice with 250 µL of viral suspension solution (10 mM Tris-HCl pH 7.4, 100 mM NaCl, 1 mM EDTA, 5% glycerol), and reverse-centrifuged at 1,100 × g for 2 minutes each time. The eluate was transferred to an ultracentrifuge tube containing 400 µL of 20% sucrose layered at the bottom. The mixture was ultracentrifuged at 45,000 rpm for 2 hours at 4°C. The viral pellet was resuspended in 70 µL of viral suspension solution and incubated overnight at 4°C with gentle mixing. Virus was aliquoted into 10 µL volumes, flash-frozen in liquid nitrogen, and stored at −80°C.

The transduction into patient tumor cells was carried out in Ultra-Low Attachment cell culture flask in D10^-^ media at a cancer cell concentration of 500,000 cells per mL. To optimize the lentiviral transduction condition, various dilutions of the viral suspension solution and a range of infection time were tested. (Fig. S1a, b). The overall infection rate (measured with GFP signal using flow cytometry) was set at ~15%, which ensures multiplicity of infection (MOI) ≤1, within 4 hours with no negative effect on subsequent cell survival (Fig. S1b, c).


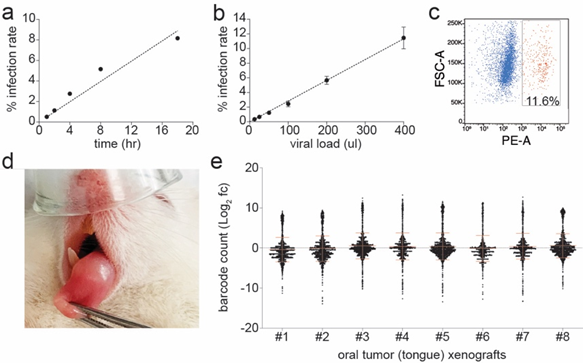


Figure S1. a) lentiviral infection rate at a set viral load versus exposure time. b) lentiviral infection rate with 4-hour exposure versus viral load. c) the infection rate measured by RFP signal using flow cytometry.

*Cell number and engraftment rate*

Purified tumor cells were obtained from subcutaneous xenografts. Various numbers of purified tumor cells in 20 µL of PBS were then injected into lingual tissue of NSG mice. The engraftment rates were recorded as shown in Table S1.

Table S1. The PDX engraftment rate versus numbers of purified tumor cells injected

| Number of tumor cells injected (in 20 µL) | Average engraftment rate (%) |
| --- | --- |
| 10,000 | 8 |
| 50,000 | 55 |
| 100,000 | 100 |
| 500,000 | 100 |

*Fraction and Allele-Specific Copy Number Estimates from Tumor Sequencing (FACETS) output for patient tumor and corresponding PDX*

*
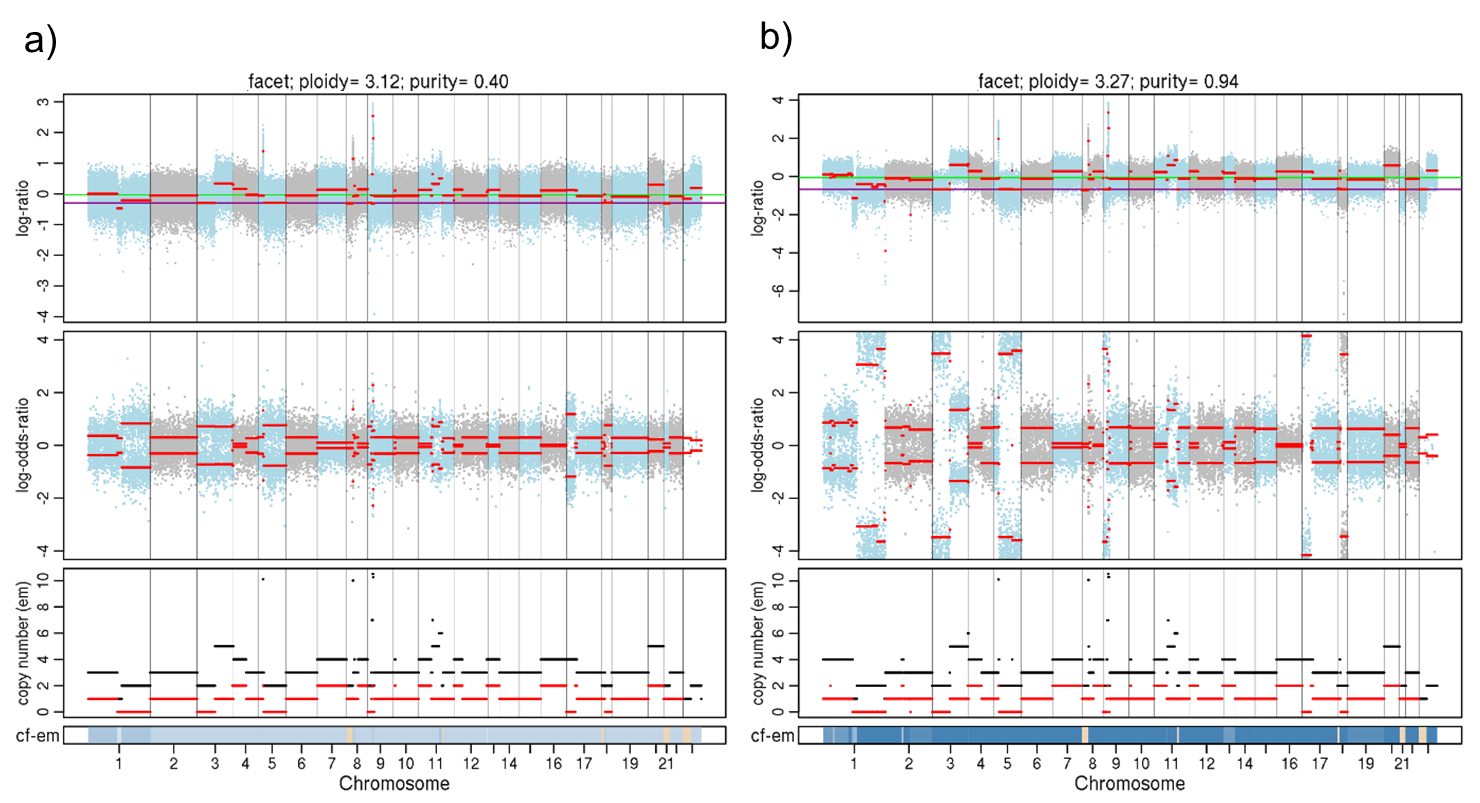
*

Figure S2. Fraction and Allele-Specific Copy Number Estimates from Tumor Sequencing (FACETS) analysis of a) Pt9 tumor and b) corresponding orthotopic PDX tumor PDX9.

Table S2. The cancer cell purity in single cell suspension without and with purification.

| Pt/PDX ID | Purity (%) | |
| --- | --- | --- |
|  | Non purified | Purified |
| Pt1/PDX1 | 33 | 86 |
| Pt2/PDX2 | 27 | 97 |
| Pt3/PDX3 | N/A | N/A |
| Pt4/PDX4 | 28 | 94 |
| Pt5/PDX5 | - | 93 |
| Pt6/PDX6 | - | 95 |
| Pt7/PDX7 | - | 83 |
| Pt8/PDX8 | 44 | 90 |
| Pt9/PDX9 | 40 | 94 |

-: all samples were purified and non-purified samples were not available for analysis
